# Single-cell and single-nucleus transcriptomics in ischemic stroke: cellular mechanisms and therapeutic implications

**DOI:** 10.3389/fneur.2026.1890630

**Published:** 2026-09-18

**Authors:** Changqing Mu, Yuchuan Ding, Alexander Weiss, Sydni Rosenfeld, Fengwu Li, Xiaokun Geng

**Affiliations:** 1 Luhe Institute of Neuroscience, Capital Medical University, Beijing, China; 2 Department of Neurology, Beijing Luhe Hospital, Capital Medical University, Beijing, China; 3 Department of Neurosurgery, Wayne State University School of Medicine, Detroit, MI, United States

**Keywords:** single-cell transcriptomics, cellular heterogeneity, neuroinflammation, cell death, neural injury and repair

## Abstract

Ischemic stroke remains a major cause of death and long-term disability and is defined by complex, dynamic pathophysiological processes involving diverse cell types and interconnected molecular networks. In recent years, single-cell and single-nucleus RNA sequencing (sc/snRNA-seq) have provided unmatched resolution for dissecting these processes. A growing body of studies has applied these approaches to ischemic stroke, generating extensive insight into cellular heterogeneity, molecular regulation, and disease-associated cell states. This review systematically integrates current evidence from sc/snRNA-seq studies, with a primary focus on brain-intrinsic mechanisms after ischemic stroke. Existing findings are organized across major pathophysiological domains, including immune and inflammatory responses, blood-brain barrier (BBB) disruption and angiogenesis, regulated cell death, metabolic dysregulation, aging and cellular senescence, and neuroregeneration and remyelination. Beyond these canonical mechanisms, we summarize emerging applications of sc/snRNA-seq in extra-CNS systems, distinct biological contexts, and stroke-related complications. We also highlight how sc/snRNA-seq has been used to investigate therapeutic mechanisms, including physical and neuromodulatory interventions, pharmacological agents, cell-based therapies, and bioengineered delivery systems. Together, this review provides a structured synthesis of how sc/snRNA-seq has advanced the understanding of ischemic stroke biology by identifying disease-relevant cell states, refining mechanistic targets, and informing future translational development.

## 1 Introduction

Ischemic stroke contributes substantially to adult mortality and long-term neurological impairment (1, 2). Epidemiological estimates suggest that the global incidence of cerebral infarction will continue to rise, with approximately 1.76 million additional cases projected by 2030 compared with 2020 levels (3). Although reperfusion therapies have improved outcomes for selected patients, their overall clinical impact remains constrained by narrow eligibility criteria and the occurrence of futile recanalization (4–6). These limitations highlight the need for a more precise understanding of the biological processes that determine injury progression, tissue repair, and functional recovery after ischemic stroke.

The pathogenesis of ischemic stroke is multifactorial, encompassing disrupted energy metabolism, oxidative stress, neuronal death, blood-brain barrier (BBB) disruption, and neuroinflammation (7). In parallel, endogenous recovery programs, including angiogenesis, neurogenesis, and remyelination, are activated (8). These injury and repair responses involve multiple cell types and are shaped by dynamic intercellular interactions, spatially restricted microenvironments, and evolving cellular state transitions across the ischemic brain. Traditional bulk-level approaches are limited in resolving this complexity because they average signals across heterogeneous cell populations and obscure cell-type-specific responses (9).

The advent of single-cell RNA sequencing and single-nucleus RNA sequencing (sc/snRNA-seq) has provided a transformative framework for overcoming these limitations by enabling transcriptomic profiling at single-cell or single-nucleus resolution (10, 11). Beyond describing gene expression changes, these approaches support systematic dissection of cellular heterogeneity, identification of disease-associated cell states, reconstruction of dynamic cellular trajectories, and inference of intercellular communication networks in the context of ischemic injury. As a result, sc/snRNA-seq has begun to reveal previously unrecognized cellular subpopulations, regulatory circuits, and cell-cell interactions that collectively shape stroke pathology.

In this review, we integrate current sc/snRNA-seq evidence to examine how cellular heterogeneity, disease-associated cell states, regulatory pathways, and intercellular communication contribute to ischemic injury and repair. We organize the literature around major pathophysiological and translational themes, emphasizing mechanistic convergence, therapeutic implications, current limitations, and future directions.

## 2 Sc/snRNA-seq technologies and analytical strategies used in ischemic stroke

The workflow and analytical framework of sc/snRNA-seq have been extensively described in previous studies (12–15). In brief, these technologies enable high-resolution gene expression profiling through isolation of individual cells or nuclei, transcript capture, sequencing, and computational analysis. In ischemic stroke research, these approaches are commonly applied to experimental models of cerebral ischemia/reperfusion (*I/R*) injury, in which transient interruption of cerebral blood flow followed by restoration of perfusion induces dynamic injury and repair responses. This setting allows investigators to examine how neural, glial, vascular, and immune cell populations respond to ischemic stress and subsequent reperfusion at single-cell or single-nucleus resolution.

After preprocessing steps such as quality control, normalization, batch correction, and feature selection, sc/snRNA-seq datasets can undergo diverse downstream analyses. Cell clustering and annotation identify major cell types and disease-associated cell states. Differential abundance and differential gene expression analyses reveal changes in cell composition and cell-type-specific transcriptional programs. Functional enrichment, gene set scoring, and metabolic pathway inference help interpret the biological meaning of these transcriptional changes. Trajectory or pseudotime analysis orders cells along inferred state transitions, such as progression from homeostatic to activated microglia or from progenitor-like to differentiated cell states, although these computational trajectories do not establish lineage relationships and require experimental validation. Other approaches, including transcription factor and gene regulatory network analysis, cell–cell communication analysis, phenotype-linked module analysis, and *in silico* perturbation analysis, can further prioritize candidate regulators, gene programs, and predicted intercellular signaling pathways involved in stroke pathology.

Together, these approaches characterize cellular composition, transcriptional states, candidate regulatory networks, and predicted intercellular interactions in the ischemic brain. Their interpretation, however, should account for the level of evidence supporting each finding, particularly when conclusions are derived from pseudotime analysis, ligand-receptor modeling, transcription factor activity inference, module scoring, or virtual perturbation rather than direct functional validation. These technologies have been applied across animal models, human brain tissues, cellular systems, and brain organoids. When combined with independent experimental validation, they can support more robust and biologically grounded interpretations of transcriptomic findings.

## 3 Pathophysiological mechanisms in the brain after ischemic stroke

Sc/snRNA-seq studies have increasingly clarified how distinct cellular populations and transcriptional programs contribute to brain-intrinsic injury and repair after ischemic stroke (Figure 1). The following sections synthesize these findings across major biological processes, highlighting how sc/snRNA-seq approaches have refined current understanding of stroke pathophysiology.

**Figure 1:**
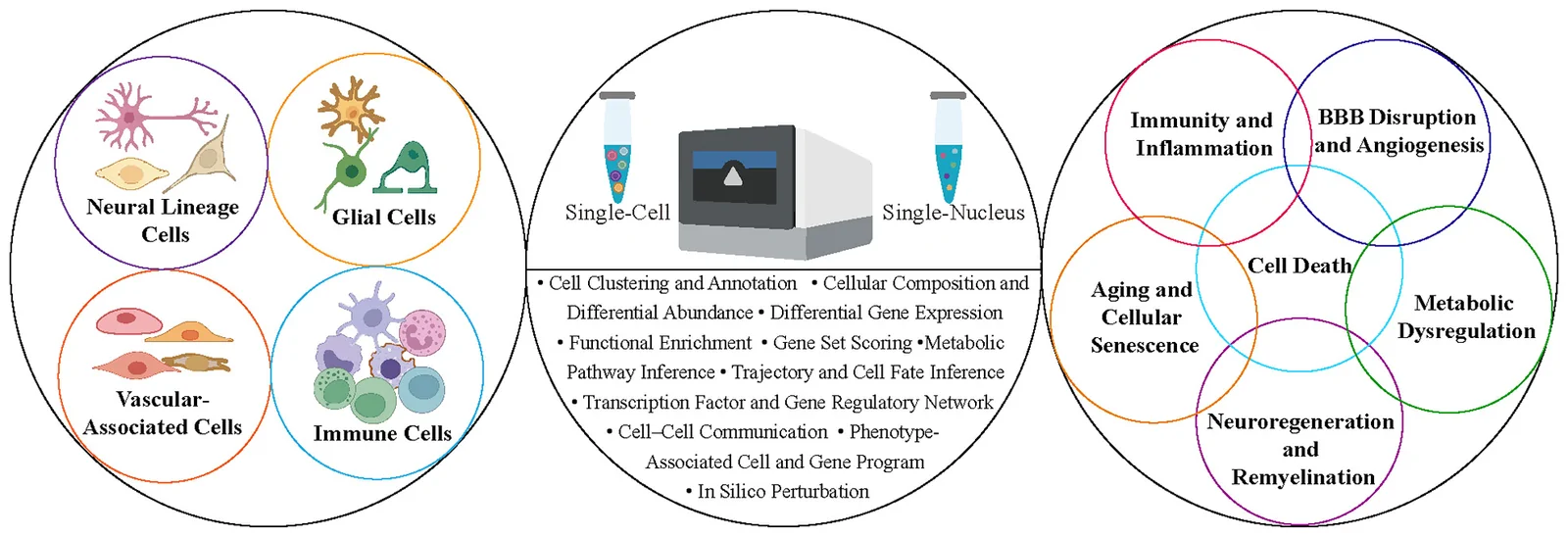
Multicellular systems-Level Sc/snRNA-seq analysis from diverse cells to interacting stroke mechanisms. Single-cell and single-nucleus RNA sequencing, combined with computational analyses such as cell clustering and annotation, differential abundance and gene-expression analysis, functional and metabolic pathway inference, trajectory analysis, gene-regulatory network reconstruction, and cellcell communication analysis (14), enables simultaneous characterization of immune, vascular-associated, glial, and neural cells. This unique and powerful capacity allows sc/snRNA-seq to move beyond single-pathway or single-cell-type analysis and instead reveal how multiple overlapping mechanisms, including inflammation, BBB disruption and angiogenesis, cell death, metabolic dysregulation, aging/senescence, and neuroregeneration/remyelination, interact within the ischemic brain. By linking diverse cell states and intercellular interactions to overlapping mechanisms of injury and repair, Sc/snRNA-seq provides a multicellular systems-level view of ischemic stroke.

A total of 101 relevant publications were identified through searches of PubMed, Web of Science, and Embase up to December 22, 2025, using combinations of terms related to ischemic stroke and single-cell or single-nucleus RNA sequencing. Original studies and secondary analyses using scRNA-seq or snRNA-seq in ischemic stroke-related human samples, animal models, cellular models, or brain organoids were considered eligible. Studies using experimental ischemic stroke models, (*I/R*) injury models, hypoxia/ischemia-related cellular systems, and stroke-related organoid platforms were included when they provided relevant single-cell or single-nucleus transcriptomic data. Duplicate records, reviews, editorials, letters, conference abstracts, books or book chapters, preprints, retracted publications, studies unrelated to ischemic stroke, studies using mixed-disease models in which ischemic stroke could not be evaluated independently, and studies without sc/snRNA-seq data were excluded. The relevant evidence was organized according to the mechanistic themes and therapeutic applications addressed in this review.

The experimental characteristics of the 101 studies included in the mechanistic synthesis are provided in Table 1. Of these studies, 96 used a single assignable experimental system or model, whereas 5 integrated multiple publicly available sc/snRNA-seq datasets generated from heterogeneous mouse stroke experiments. Among the 96 studies with an assignable system or model, 83 used mice, 9 used rats, 2 analyzed post-stroke human brain tissue, 1 used a common marmoset, and 1 used a human iPSC-derived brain organoid. Age and sex were summarized for the 93 *in vivo* animal studies. Among these studies, 82 used young or adult animals, 4 compared young and aged animals, 1 included neonatal, young/adult, and aged animals, and 5 lacked sufficient age information for classification. Regarding sex, 82 studies used only male animals, 8 used both males and females, and 3 lacked sufficient information; no study used only female animals. Among the 93 in vivo animal studies, 75 used tMCAO, 10 used pMCAO, 5 used ET-1-induced stroke, and 3 used photothrombotic stroke. The remaining three studies analyzed human ischemic stroke tissue (*n* = 2) or used a human brain organoid hypoxia model (*n* = 1).

**Table 1:** Experimental characteristics of the sc/snRNA-seq studies included in the mechanistic synthesis.

Characteristic	Number of studies
Species/model system	
Mouse	83
Rat	9
Human post-stroke tissue	2
Common marmoset	1
Human iPSC-derived brain organoid	1
NA (multi-dataset integration)	5
Age (93 *in vivo* animal studies)	
Young or adult animals	83
Young-versus-aged comparison	4
Multiple life stages (neonatal, young adult, and aged)	1
Not reported/unclear	5
Sex (93 *in vivo* animal studies)	
Male only	82
Female only	0
Both sexes	8
Not reported/unclear	3
Ischemic stroke model (93 *in vivo* animal studies)	
tMCAO	75
pMCAO	10
ET-1-induced stroke	5
Photothrombotic stroke	3

Five studies performed integrated analyses of multiple publicly available sc/snRNA-seq datasets derived from heterogeneous mouse stroke experiments; therefore, no single species/model system, age, sex, or stroke model could be assigned to these studies, and the corresponding entries were classified as not applicable. Age, sex, and ischemic stroke models were summarized for the 93 in vivo animal studies. Age categories were interpreted according to the definitions used in the original studies and do not indicate equivalent absolute ages across species. “Not reported/unclear” denotes information that was absent, insufficiently specified, internally inconsistent, or unavailable for classification. NA, not applicable; tMCAO, transient middle cerebral artery occlusion; pMCAO, permanent middle cerebral artery occlusion; ET-1, endothelin-1; iPSC, induced pluripotent stem cell.

Across these experimental contexts, sc/snRNA-seq studies have provided cell-type-resolved insights into six major aspects of ischemic stroke pathophysiology: immunity and inflammation, blood–brain barrier disruption and angiogenesis, cell death, metabolic dysregulation, aging and cellular senescence, and neural regeneration and remyelination. Representative cellular states, molecular programs, and intercellular alterations identified across these mechanistic domains are summarized in Figure 2.

**Figure 2:**
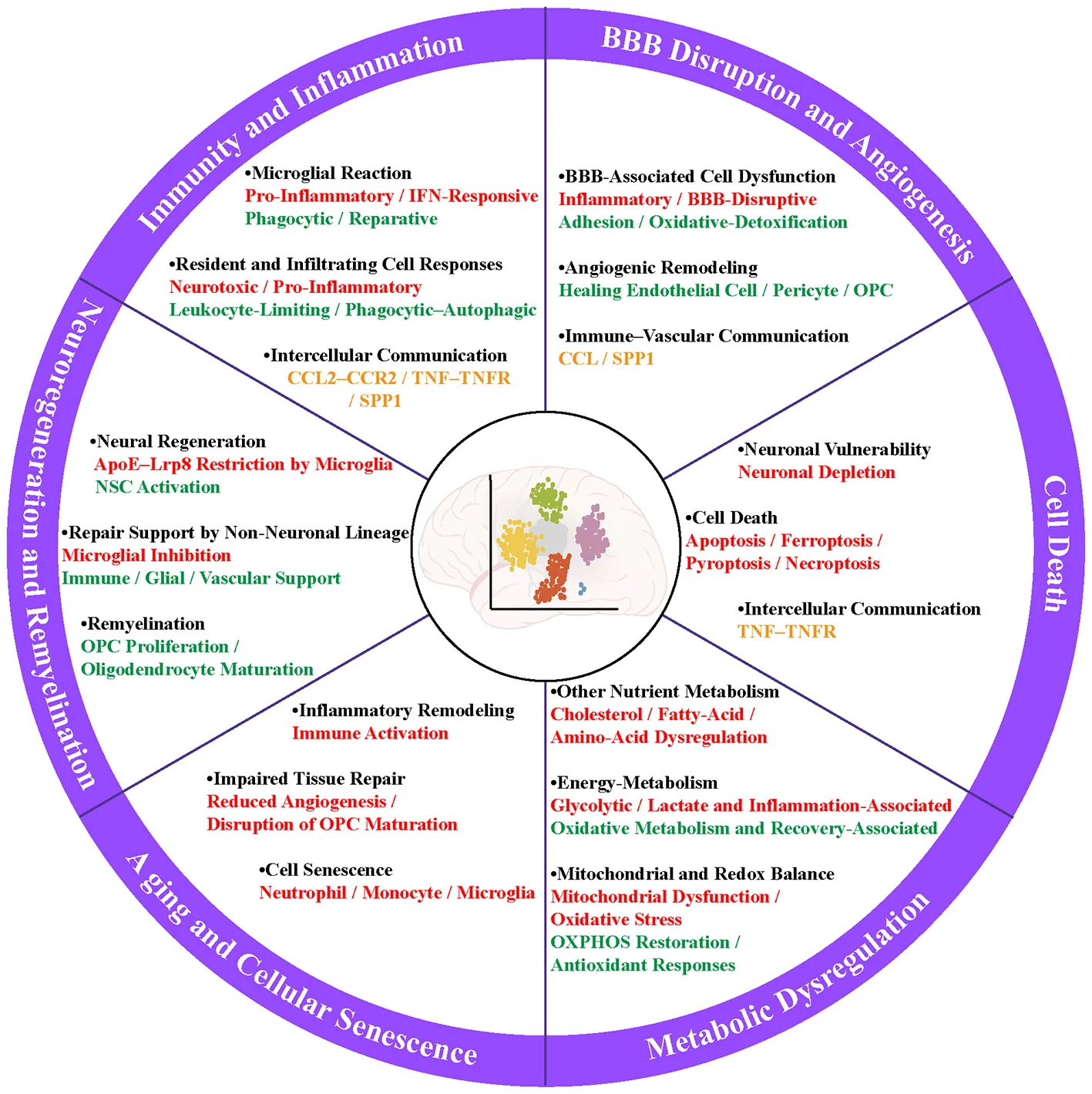
Overlapping and bidirectional mechanisms revealed by sc/snRNA-seq in ischemic stroke. Within each major stroke-related mechanism, sc/snRNA-seq reveals that cellular responses are not equally harmful or beneficial, but often include simultaneous detrimental (red), neuroprotective (green), and context-dependent or transitional effects (orange). Inflammation, BBB disruption/angiogenesis, cell death, metabolic dysregulation, aging/senescence, and neuroregeneration/remyelination each involve interacting cell states and pathways that may promote injury, support repair, or exert neutral/transitional roles depending on cell type, timing, and microenvironment. This system-level view highlights how ischemic stroke pathophysiology emerges from overlapping and interconnected mechanisms across multiple cellular compartments.

### 3.1 Immunity and inflammation

Immune and inflammatory responses are integral to all stages of ischemic stroke and involve dynamic interactions among resident brain cells, infiltrating immune cells, and vascular compartments (16, 17). Sc/snRNA-seq has substantially advanced our understanding of this process by showing that post-stroke inflammation is not simply driven by a uniform microglial response but by multiple disease-associated cellular states that evolve over time.

One major insight from sc/snRNA-seq studies is that post-stroke microglial activation is not adequately captured by the traditional M1/M2 polarization paradigm. Early single-cell atlases of the ischemic hemisphere identified diverse microglial and immune-cell populations after middle cerebral artery occlusion/reperfusion (MCAO/R), including pro-inflammatory, interferon-responsive, proliferative, phagocytic/anti-inflammatory, and neuropeptide-associated microglial subsets (18–25). These findings indicate that inflammatory and reparative programs can coexist within the ischemic brain and that microglial responses are better understood as a spectrum of dynamic states rather than binary phenotypes. At the molecular level, activated microglia show increased expression of inflammatory genes such as Cd72, Cd74, and Adam8, together with reduced expression of homeostatic regulators such as Plxdc2 (26–29). Integrated transcriptomic and trajectory analyses further implicate Rela-centered transcriptional regulation (30), STING–type I interferon signaling (31), Ftl1/Fth1-related ferritin programs (32), Slc2a5-associated repair pathways (33), and Lrg1-associated pro-inflammatory programs (34) in shaping inflammation-related microglial state transitions.

Importantly, sc/snRNA-seq studies also show that post-stroke inflammation is not restricted to microglia. Astrocytes exhibit inflammatory and potentially neurotoxic transcriptional programs involving CEBPB–CCL2/ADM, STAT3, and PI3K–AKT signaling (35–37), while nonhuman primate snRNA-seq data identified RTN4A^+^ astrocytes associated with regulation of leukocyte responses (38). Neurons may also actively participate in immune signaling. Early Cebpb induction in an ischemic stroke-specific neuronal subpopulation with immune-related signaling features suggests that neurons are not merely passive targets of inflammatory injury but may contribute to neuroimmune communication (39).

Peripheral and border-associated immune cells represent another key layer of the post-stroke inflammatory response. Sc/snRNA-seq studies have identified enrichment of monocyte-derived cells, neutrophils, and macrophage subsets in the ischemic brain (18). Mrc1^+^ border-associated macrophages may promote neuroinflammation through the Stat3–Tnf axis (40), whereas Foxp3^+^ macrophages show phagocytic and autophagy-related programs (41). Clec4D may contribute to neutrophil activation (42), and neutrophil responses are highly dynamic, with early expansion of Mmp8^+^ neutrophils showing high NET-related transcriptional activity and later persistence of neutrophils expressing S100a8/a9, Mmp-9, and Cxcr2, suggesting that infiltrating immune cells may sustain inflammatory injury beyond the acute phase (43, 44).

Cell–cell communication analyses provide a framework for integrating these cellular findings. Ligand–receptor studies frequently identify microglia as predicted communication hubs linked to neurons, astrocytes, endothelial cells, and infiltrating immune cells through pathways such as CCL2-CCR2 and TNF-TNFR (18, 45–47). In addition, Il2ra-high Cd8^+^ *T* cell-endothelial interactions, and SPP1- and TNF-associated monocyte/macrophage-endothelial signaling suggest extensive immune–immune and immune–vascular coordination after stroke (48, 49).

Taken together, sc/snRNA-seq studies have reframed post-stroke inflammation as a dynamic, multicellular process involving resident microglia, astrocytes, neurons, infiltrating immune cells, and vascular compartments. Rather than simply revealing “many cells and many pathways,” these studies suggest that ischemic inflammation is organized around several convergent themes, such as microglial state diversification, activation of inflammatory and interferon-related programs, recruitment of peripheral immune cells, and increased predicted immune–vascular communication. However, current research remains disproportionately focused on microglia and peripheral immune cells, while the immunomodulatory roles of other brain-resident cells and the integration of neuroimmune with neurovascular processes remain incompletely defined. Addressing these gaps will be essential for developing more precise and effective immunomodulatory strategies for ischemic stroke.

### 3.2 Blood–brain barrier disruption and angiogenesis

The BBB maintains cerebral homeostasis and limits excessive neuroinflammation, while angiogenesis supports restoration of oxygen and nutrient delivery after ischemic stroke. Both processes are highly sensitive to (*I/R*) injury and involve coordinated responses among endothelial cells, pericytes, astrocytes, immune cells, and other vascular-associated cell types (50–54). Sc/snRNA-seq has provided greater resolution for understanding how BBB disruption and vascular repair unfold at the cellular and molecular levels after stroke.

Single-cell studies have shown that BBB injury is not limited to endothelial dysfunction but reflects coordinated remodeling of multiple barrier-associated cell types. In ischemic brain tissue, an endothelial population characterized by high expression of Adamts4, Upp1, and Timp1 is expanded after stroke, suggesting a potential role in BBB breakdown (18). Pericytes undergo early inflammatory remodeling, with an ischemia-specific Il6/Ccl2-expressing subpopulation emerging within 12–24 h after stroke (55). Slc22a8, a BBB endothelial-enriched gene, is downregulated after stroke, while astrocytes show increased expression of barrier-injury-associated genes such as Mmp9 and Mmp12 in delayed thrombolysis models (37, 56). These findings suggest that post-stroke BBB disruption arises from convergent endothelial, mural-cell, and astrocytic responses rather than from a single vascular cell type.

Sc/snRNA-seq studies have also identified candidate regulatory pathways that may influence BBB integrity. Lrg1 is upregulated across major BBB-associated cell types after brain I/R injury. In Lrg1 knockout mice, expression of genes related to ribosome function, cell adhesion, and oxidative detoxification is increased, as are levels of adhesion-associated proteins such as claudin-11, integrin-β5, annexin A2, and protocadherin 9, suggesting that Lrg1 may contribute to BBB destabilization by affecting cell adhesion and oxidative stress responses (34). In addition to these intrinsic vascular changes, ligand–receptor analyses predict enhanced inflammatory communication between myeloid and endothelial cells, particularly through CCL and SPP1 pathways, suggesting that immune activation may amplify vascular inflammation and barrier injury (57).

In contrast to BBB breakdown, angiogenesis represents an endogenous repair response that begins within hours to days after stroke and evolves over time (58). ScRNA-seq performed 1 day after I/R identified a “healing” endothelial cell subset with pro-angiogenic features, high inferred stemness, and Xbp1 as a potential regulatory factor (59). Interestingly, although Lrg1 has been associated with BBB disruption, Lrg1^+^ endothelial cells in a permanent MCAO model were enriched for vascular-remodeling pathways at day 3, suggesting that Lrg1 may have context-dependent roles in both vascular injury and repair (60). Pericyte plasticity may also contribute to angiogenesis, as Tbx18^+^ pericytes were positioned along an inferred endothelial-like trajectory by day 3 after stroke, and a rare transient pericyte subpopulation was enriched for angiogenesis, cell migration, and wound-healing pathways (61, 62). Beyond vascular cells, Vegfa-expressing OPCs with hypoxia-related signatures may also participate in angiogenic remodeling, highlighting the multicellular nature of post-stroke vascular repair (63).

Taken together, these findings indicate that BBB disruption and angiogenesis after stroke are tightly linked but biologically distinct vascular processes. BBB injury is characterized by endothelial dysfunction, inflammatory pericyte activation, astrocytic MMP induction, Lrg1-associated barrier destabilization, and myeloid–endothelial inflammatory signaling. Concurrently, vascular repair involves pro-angiogenic endothelial states, pericyte plasticity, vascular-remodeling programs, and possible support from OPCs. Thus, sc/snRNA-seq has shifted our understanding of post-stroke vascular pathology from a simple model of barrier leakage to a dynamic multicellular process involving both injury and repair. However, most current studies remain limited by single-time-point analyses and incomplete functional validation. Future studies integrating longitudinal sc/snRNA-seq, spatial transcriptomics, and experimental validation will be important for defining therapeutic windows and clarifying how endothelial, mural, glial, and immune-cell interactions determine BBB integrity and angiogenic recovery after stroke.

### 3.3 Cell death

Cell death is a central pathological process in ischemic stroke and includes both nonregulated and regulated mechanisms, such as necrosis, apoptosis, necroptosis, autophagy-associated cell death, ferroptosis, and pyroptosis (64). These processes drive the irreversible loss of neurons, glial cells, and other brain cell types, thus contributing to neurological disability and mortality after stroke. Recent sc/snRNA-seq studies have begun to define stroke-induced cell death at higher resolution by identifying vulnerable cell populations, death-associated transcriptional programs, and interactions between dying or stressed cells and the inflammatory microenvironment.

One major insight from snRNA-seq is the marked vulnerability of neuronal populations after ischemic injury. Stroke-affected brain regions show substantial depletion of both glutamatergic and GABAergic neurons, including parvalbumin-, vasoactive intestinal polypeptide-, and somatostatin-expressing GABAergic neurons, as well as glutamatergic intratelencephalic neurons in cortical layers 2/3 and 6 (65, 66). Although these studies did not directly distinguish specific death pathways, they are consistent with marked and selective neuronal loss after stroke. Gene set-based analyses further suggest that ischemic injury is associated with multiple regulated cell-death programs across diverse cell types. In MCAO/R models, transcriptional signatures of apoptosis, necroptosis, autophagy, pyroptosis, and ferroptosis are concurrently elevated, while additional studies have reported higher scores for cuproptosis and disulfidptosis (67–74). These findings indicate that post-stroke cell death is unlikely to be explained by a single dominant pathway; rather, different cell types may engage overlapping death-associated and stress-response programs during injury progression.

Several candidate molecular mechanisms have been implicated in these death-related programs. Ferroptosis-related analyses have implicated Ftl1 and Fth1 as important genes distinguishing ischemic from sham conditions, while Bach1 has been proposed as a potential regulator of ferroptosis in astrocytes (32, 75). Brain organoid models further support the high sensitivity of human neural cells to hypoxic stress. In these models, cortical excitatory neurons rapidly upregulated apoptosis-related genes, including CDC42, SRPK2, and CDC34, within 1 h of hypoxia, followed by increased expression of canonical apoptotic markers such as JUN, LGALS1, and DDX3X by 8 h. Neural stem cells also exhibited apoptosis-related transcriptional programs by 8 h, suggesting that ischemic stress affects both mature neurons and neurogenic populations (35). Other stress-response pathways have also been linked to cell death after stroke, including increased autophagy scores associated with higher H_2_S levels in microglia, endothelial cells, and astrocytes, as well as Manf as a potential upstream regulator of pyroptosis (67, 72).

Sc/snRNA-seq analyses suggest that cell death is closely connected to post-stroke inflammation. Microglial states associated with cuproptosis and disulfidptosis show increased predicted communication with immune and stromal cells, mainly through TNF–TNFR signaling (73, 74). These analyses suggest that death-associated transcriptional programs may reflect cellular injury and be associated with inflammatory amplification within the ischemic brain.

In summary, sc/snRNA-seq studies have reframed our understanding of post-stroke cell death from a pathway-specific model to a broader view of dynamic, multicellular injury states. Neuronal populations show marked vulnerability, while transcriptional signatures associated with multiple regulated cell-death programs, including apoptosis, necroptosis, autophagy-associated cell death, pyroptosis, ferroptosis, cuproptosis, and disulfidptosis, are elevated across different cell types. Candidate regulators and pathways, including Ftl1/Fth1, Bach1, Manf, and apoptosis-associated genes in hypoxic organoids, provide entry points for future mechanistic studies. However, the application of sc/snRNA-seq to cell death remains limited by technical challenges, including preferential capture of viable or recoverable cells and reduced recovery of fragile neurons during scRNA-seq dissociation (76, 77). Future studies integrating snRNA-seq, spatial transcriptomics, temporal profiling, and functional validation may be better able to distinguish irreversible cell death from potentially reversible stress states and to clarify how cell-death programs interact with inflammation and tissue repair after ischemic stroke.

### 3.4 Metabolic dysregulation

Metabolic dysregulation is a hallmark of ischemic stroke and encompasses mitochondrial dysfunction, enhanced glycolysis, impaired oxidative phosphorylation, altered redox balance, and abnormal lactate metabolism (78). These changes contribute to cellular injury, impaired repair, and poor functional outcomes. For example, stress hyperglycemia in stroke patients without prior diabetes has been associated with greater stroke severity and worse 90-day outcomes after mechanical thrombectomy (79). Sc/snRNA-seq studies have begun to clarify how metabolic responses differ across cell types and how these changes may shape post-stroke injury and recovery.

Among brain cell types, microglia have been the most extensively studied in post-stroke metabolic reprogramming. Single-cell analyses suggest that inferred microglial metabolic programs vary according to injury location and disease stage. Microglia in the ischemic core show greater enrichment of glycolysis-related transcriptional programs, while those in the penumbra are enriched for citrate cycle and oxidative phosphorylation pathways (80, 81). Glycolysis-high microglia express higher levels of the metabolic risk gene P4ha1 and show increased predicted communication with surrounding cells (82). Other studies identified the E2f1–Cdk5 axis as a potential regulator of microglial mitochondrial dysfunction, while deletion of *Nhe1* or *Lrg1* increased oxidative phosphorylation-related genes, suggesting that ion transport and Lrg1 signaling may influence microglial energy metabolism after stroke (34, 83–86).

Microglial metabolic adaptation also involves redox, lipid, choline, and lactate-related pathways. Stroke-associated microglia show upregulation of the Prdx1–Srxn1–Txn1 antioxidant pathway, which may represent a compensatory response to oxidative stress (87). In the chronic phase, microglial subsets display increased cholesterol metabolism, phagocytosis, and inflammatory gene expression, with pseudotime analysis suggesting that lipid metabolic changes may precede inflammatory activation (88). Choline metabolism-related signatures are reduced after ischemic stroke, particularly in microglia, T cells, and endothelial cells (89). Lactate metabolism has also emerged as an important axis, as an acute-phase microglial subset enriched for lactate transport genes was linked to inflammatory responses, and Ldhb, expressed mainly in microglia, astrocytes, and macrophages, was associated with stroke risk in single-cell transcriptomic and Mendelian randomization analyses (90, 91).

Beyond microglia, sc/snRNA-seq studies have identified metabolic disturbances in neurons, astrocytes, endothelial cells, and peripheral immune cells. In a photothrombotic model, most non-immune brain cells showed reduced enrichment of aerobic metabolic pathways one month after stroke (66). Astrocytes exhibited transient reductions in transcriptional signatures of glycolysis, the pentose phosphate pathway, the TCA cycle, and fatty acid metabolism at 12 h after stroke, with partial recovery by 24 h, suggesting partial restoration of metabolic transcriptional programs after reperfusion (92). In brain organoid models exposed to hypoxia, astrocyte subtypes downregulated oxidative phosphorylation and mitochondrial ATP synthesis genes, including NDUFB9, NDUFA13, and NDUFB8 (35). Endothelial cells showed elevated oxidative stress scores, reduced enrichment of antioxidant pathways, including glutathione, niacin, and ascorbate metabolism, and reduced amino acid uptake-related metabolic modules, which may impair vascular repair and BBB integrity (57, 92). In peripheral immune cells, Lcp1-high monocytes/macrophages were enriched for fatty acid metabolism pathways during the acute phase after ischemia (93).

Together, these findings show that post-stroke metabolic dysregulation extends beyond energy failure alone and involves coordinated changes in glycolysis, mitochondrial oxidative phosphorylation, antioxidant defense, lipid metabolism, choline metabolism, lactate handling, and amino acid metabolism across multiple cell types. Current evidence is most developed for microglia, in which metabolic reprogramming appears closely linked to inflammation, oxidative stress, and intercellular communication. However, the metabolic trajectories of neurons, astrocytes, endothelial cells, and other vascular-associated cells remain less well defined. In addition, most studies rely on transcription-based pathway inference, such as GSVA or scMetabolism, rather than direct measurement of metabolic flux. Future studies integrating sc/snRNA-seq with spatial transcriptomics, metabolomics, scFEA or Compass-based flux modeling, and functional validation may help define the actual metabolic changes and their biological consequences after ischemic stroke.

### 3.5 Aging and cellular senescence

Aging is a major risk factor for ischemic stroke and is strongly associated with worse outcomes (94). At the same time, stroke itself may promote cellular senescence, which may contribute to persistent neuroinflammation, impaired repair, and long-term functional decline (95). Recent scRNA-seq studies have begun to clarify how aging reshapes post-stroke cellular responses and how senescence-associated programs may contribute to poor recovery.

A major finding from single-cell studies is that aging amplifies and prolongs post-stroke immune activation. In aged mice subjected to transient MCAO, scRNA-seq of Cd45^+^ immune cells revealed increased proportions of monocytes/macrophages, neutrophils, and dendritic cells on day 3 after stroke, followed by increased microglia and lymphocyte proportions by day 14 (96, 97). In the aged post-stroke brain, microglia exhibited a neutrophil-like transcriptional phenotype (96). These findings suggest that aging also alters immune-cell states.

Aging-associated changes are not limited to immune cells but also affect vascular and glial repair programs. Endothelial cells in aged mice after stroke show stronger interferon responses, whereas the pro-angiogenic Lrg1^+^ endothelial subset is reduced, accompanied by downregulation of Aplnr-associated angiogenic signaling (60). Aging also disrupts oligodendrocyte lineage progression, as the OPC3 transitional oligodendrocyte precursor state shows impaired maturation into myelinating oligodendrocytes after stroke (60). These findings indicate that aging may worsen stroke outcomes not only by intensifying inflammation but also by weakening angiogenesis and remyelination.

At the molecular and intercellular levels, aging appears to shift the post-stroke brain toward chronic inflammation and impaired repair. In a permanent MCAO model, stroke and aging synergistically increased Ifi27l2a expression in microglia, suggesting a potential role in chronic neuroinflammation (97). Cell–cell communication analyses further predicted reduced reparative signaling, including VEGFA, GDF15, FN1, and SPP1 pathways, between microglia/macrophages and endothelial or oligodendrocyte lineage cells (60). In addition, senescence-related signatures were detected in neutrophils at later stages after stroke, and elevated senescence scores were observed in microglia and monocytes as early as day 1 (98, 99).

These findings suggest that aging worsens post-stroke pathology through a combination of amplified immune activation, enhanced oxidative-stress and senescence-associated programs, impaired vascular repair, and disrupted oligodendrocyte maturation. ScRNA-seq has provided important insights into these age-related cellular changes, but current studies remain limited in number, largely descriptive, and mainly focused on immune responses. Future studies using aged stroke models, longitudinal sampling, spatial transcriptomics, and functional validation could determine how aging and senescence interact across immune, vascular, glial, and neuronal compartments. Such work may help identify age-adapted or senescence-targeted therapeutic strategies for ischemic stroke.

### 3.6 Neuroregeneration and remyelination

Neuronal loss and demyelination are major consequences of ischemic stroke and contribute directly to neurological dysfunction. However, ischemic injury also activates endogenous repair programs, including neurogenesis, synaptic remodeling, axonal repair, and remyelination (100). Sc/snRNA-seq has helped define these repair responses at single-cell or single-nucleus resolution, showing that post-stroke repair responses involve not only neurons and oligodendrocyte lineage cells but also microglia, astrocytes, vascular cells, pericytes, and innate immune cells.

Single-cell studies indicate that neuroregeneration after stroke involves both activation of neural progenitor populations and remodeling of surviving neurons. In the subventricular zone, increased proportions of neural stem cells (NSCs), transit-amplifying cells, and neuroblast populations are consistent with activation of endogenous neurogenesis after injury (101). In human post-stroke hippocampal tissue, snRNA-seq inferred a trajectory from quiescent to active NSCs, accompanied by increased neuronal and glial lineage gene expression, suggesting potential roles for these cells in repair and remyelination (102). In the chronic phase, GABAergic and glutamatergic neurons within the infarct core were enriched for synaptic plasticity-related pathways, suggesting that surviving neurons may retain transcriptional programs associated with circuit remodeling and functional recovery (66).

Neuroregeneration is shaped by signals from non-neuronal cells. Several microglial states appear to support repair: Spp1^+^ axon tract-associated microglia may promote neuronal repair through TGF-β and GAS6 signaling, while Trem2^+^Igf1^+^ microglia may support neuronal recovery through neurotrophic-factor expression (103, 104). Integrated scRNA-seq, bulk RNA-seq, and ATAC-seq analyses further identified a Pla2g2e–Padi4 axis in peri-infarct neurons associated with pro-regenerative gene expression through histone citrullination (105). However, microglia may also restrict repair. In the SVZ, increased ApoE–Lrp8 signaling between microglia and neural stem/progenitor cells inhibited neuroblast growth and limited neuroregeneration (106). These findings suggest that microglia can have both pro-repair and anti-repair effects depending on their state, location, and timing after stroke.

Other cell types similarly contribute to the post-stroke repair environment. Stem-like OPC1 cells in human post-stroke cortex were enriched for pathways related to synaptic signaling, neuronal development, and dendritic growth (107). Type 2 innate lymphoid cells (ILC2s) promoted NSPC proliferation and neuroblast differentiation, while Nestin^+^Ascl1^+^ astrocytes showed high transcriptional entropy and developmental potential after ischemic injury (36, 108). Additional cell populations may participate in repair: NG2 glia displayed neural progenitor-like features, and vascular smooth muscle cells upregulated Ngf, suggesting paracrine support for neural recovery (34, 109).

Remyelination after stroke is driven primarily by oligodendrocyte-lineage cells and is regulated by intercellular communication. OPCs rapidly shifted from quiescent to proliferative states within 3 days after transient MCAO, while mature oligodendrocytes at day 28 were enriched for axonal ensheathment, extracellular matrix remodeling, and Wnt signaling pathways (60, 66). In a brainstem stroke model, OLG8 oligodendrocytes showed hypoxia- and MAPK-related programs and appeared to interact with IN2 interneurons through EGF signaling, suggesting coordinated neuroprotective and remyelinating responses (110). Microglia also regulate white matter repair; microglia-specific deletion of Slc9a1 expanded myelin-supporting MG3 microglia and promoted OL5 oligodendrocytes with enhanced phagocytic and lactate-shuttling functions, potentially through Psap–Gpr37 signaling (84). Similarly, Spp1^+^Ccnb1^+^ microglia were inferred to communicate with OPCs and potentially regulate remyelination in a pontine stroke model (111).

Taken together, these findings show that post-stroke neuroregeneration and remyelination are coordinated multicellular processes rather than neuron- or oligodendrocyte-restricted events. Sc/snRNA-seq studies have identified activation of NSCs and neuroblasts, synaptic plasticity programs in surviving neurons, pro-regenerative and inhibitory microglial signals, repair-associated astrocyte and pericyte states, and oligodendrocyte lineage responses involved in myelin restoration. Two important questions remain unresolved. First, whether newly generated neurons after stroke meaningfully contribute to higher-order functions such as learning, memory, and emotion remains unclear. Second, because oligodendrocytes are more abundant in the human brain than in rodents, nonhuman primate and human-relevant models may be needed to better define post-stroke myelin dynamics (112, 113). Future studies integrating longitudinal sc/snRNA-seq, spatial transcriptomics, lineage tracing, and functional validation could determine the specific regenerative programs that are functionally reparative and therapeutically actionable after ischemic stroke.

## 4 Mechanisms of therapeutic interventions

Despite substantial progress in basic stroke research, the clinical translation of neuroprotective strategies remains challenging, and clinical trial outcomes have been inconsistent (114). With the rapid development of single-cell and single-nucleus transcriptomics, sc/snRNA-seq has increasingly been applied in preclinical studies to explore the cellular and molecular mechanisms underlying therapeutic interventions in ischemic stroke. Compared with conventional approaches that mainly evaluate treatment efficacy at the tissue or behavioral level, sc/snRNA-seq enables treatment-associated cellular responses to be resolved at single-cell or single-nucleus resolution, providing a framework for characterizing changes in cellular states and inferred intercellular interactions across the injured brain.

Although reperfusion therapy improves outcomes, many patients with acute ischemic stroke continue to experience poor outcomes, particularly those with large infarct cores, highlighting the need for adjunctive neuroprotective strategies (115). Many therapeutic strategies, including physical and neuromodulatory interventions, pharmacological agents, traditional herbal formulations, cell-based therapies, and bioengineered delivery systems, have shown potential benefit in experimental stroke models. However, their cellular targets and downstream mechanisms remain incompletely defined. Sc/snRNA-seq provides an opportunity to clarify these mechanisms by identifying cell-type-specific responses, candidate molecular targets, and signaling pathways that may guide their future refinement. These studies were conducted exclusively in preclinical models and are summarized in Table 2 by therapeutic class.

**Table 2:** Sc/snRNA-seq findings from preclinical studies of therapeutic interventions in Ischemic stroke.

**Treatment**	Key findings	Mechanism
Physical and neuromodulatory interventions		
Low-intensity focused ultrasound	Angiogenesis by astrocyte Hmgb1↑; Synaptic plasticity by astrocyte Camk2n1↓ (116)	Angiogenesis; neuroregeneration
High-frequency rTMS	Expansion of pericytes; Increased pericytes; enhanced endothelial communication via CCL/TGF-β; Pericyte-VSMC differentiation (117)	BBB Disruption; angiogenesis
Acupuncture	Modulation of macrophage activity and Ras signaling regulation (118)	Immunity and inflammation
Pharmacological and small-molecule therapies		
11-keto-β-boswellic acid + Z-Guggulsterone	Spp1-driven microglia/astrocyte neuroprotective reprogramming (119)	Immunity and inflammation
PLX5622	Expansion of reparative endothelial subsets; Suppression of inflammatory microglia (120); Expansion of Mafb-driven repair-associated macrophages enriched in lipid metabolism, myelin repair, angiogenesis pathways (121)	Immunity and inflammation; Remyelination; angiogenesis; metabolic Dysregulation
Dexmedetomidine	Expansion of reparative endothelial subsets; Suppression of inflammatory microglia (122)	Angiogenesis; immunity and inflammation
QO-83	Restoration of neuronal potassium voltage-gated channel subfamily Q channels; Microglia polarization toward anti-inflammatory phenotype (123)	Neuroregeneration; immunity and inflammation
Soluble PD-L1	Shift of circulating monocytes from inflammatory phenotype to anti-inflammatory phenotype (124)	Immunity and inflammation
AAV-VEGF-C Intracerebrospinal Delivery	Activation of BDNF and tyrosine kinase signaling across neurons, astrocytes, endothelial cells (125)	Immunity and inflammation
Traditional Chinese medicine		
Huanglian Jiedu decoction	Reduction of T cell metabolic activity; Suppression of pro-inflammatory double-negative T cells (126)	Immunity and inflammation; Metabolic dysregulation
Dalbergia odorifera leaf extract	Restoration of oxidative phosphorylation; Promotion of M2 microglia polarization (127)	Metabolic dysregulation; immunity and inflammation
Dengzhan Shengmai Capsule	Attenuation of neuronal inflammation and apoptosis by downregulation of Ifitm3 and Vim (128)	Immunity and inflammation; Cell Death
Buyang Huanwu decoction	Endothelial transcriptional restoration; Hippo pathway activation; Resistance to apoptosis (129)	BBB Disruption and angiogenesis; cell death
Cell-based and regenerative therapies		
Bone marrow-derived macrophage transplantation	Upregulation of homeostatic microglia; and axon tract-associated microglia (103)	Neuroregeneration; immunity and inflammation
MSC-derived migrasomes	Thymic epithelial cell expansion and differentiation toward cortical thymic epithelial cells and medullary thymic epithelial cell II (130)	Immunity and inflammation
NPC transplantation	Differentiation of radial glial cells and proliferative progenitors into excitatory neurons (131)	Neuroregeneration
Nanotechnology and bioengineered delivery systems		
ROS-responsive liposomes for puerarin delivery	Expansion of reparative endothelial subsets; Suppression of inflammatory microglia (132)	Angiogenesis; immunity and inflammation
Neutrophil Membrane-Camouflaged Polyprodrug Nanomedicine (FTY720)	Cebpb downregulation–mediated microglia reprogramming toward anti-inflammatory state (133)	Immunity and inflammation
Aged neutrophil membrane-coated CGRP nanoparticles for its targeted delivery to bone marrow	Expansion of G2/M4 myeloid subsets; Enhanced anti-inflammatory capacity of myeloid-derived suppressor cells (134)	Immunity and inflammation
Olig2 mRNA delivery (LNP)	Accelerated OPC maturation; Enhanced neuroplasticity by activation of LTP/LTD and calcium signaling pathways (135)	Neuroregeneration and remyelination
OMV@PGZ (pioglitazone vesicles)	Reduced neuron–oligodendrocyte interaction; activation of anti-inflammatory and repair-associated oligodendrocytes by activation of Pou2f1 and Nrf1 (136)	Immunity and inflammation; Remyelination; neuroregeneration
QG-EVs	Enhanced neuron–oligodendrocyte interactions; Reduced inflammatory signaling between neuron and macrophage (137)	Neuroregeneration; immunity and inflammation

This table categorizes therapeutic interventions for ischemic stroke (physical/neuromodulatory, pharmacological, traditional Chinese medicine, cell based, and nanotechnological approaches) and highlights representative mechanistic insights derived from sc/snRNA seq, covering key processes such as angiogenesis, immunity/inflammation, metabolic regulation, neuroregeneration, and remyelination. AAV, adeno-associated virus; EVs, extracellular vesicles; FTY720, fingolimod hydrochloride; LNP, lipid nanoparticle; LTP/LTD, long-term potentiation / long-term depression; MSC, mesenchymal stem cell; NPC, neural progenitor cell; OMV@PGZ, outer membrane vesicle–loaded pioglitazone; OPC, oligodendrocyte precursor cell; PLX5622, colony-stimulating factor 1 receptor inhibitor; QG-EVs, quercetin-loaded extracellular vesicles; QO-83, a Kv7/KCNQ channel opener; ROS, reactive oxygen species; rTMS, repetitive transcranial magnetic stimulation; VSMC, vascular smooth muscle cell.

These studies highlight the growing value of sc/snRNA-seq for uncovering the cellular and molecular mechanisms of therapeutic interventions in ischemic stroke. By enabling cell-type-specific profiling of gene expression and signaling dynamics, these approaches suggest that different treatment modalities may converge on key biological processes, including neuroprotection, immune modulation, angiogenesis, BBB maintenance, and neuroregeneration. However, several translational limitations should be recognized. First, most available studies have been performed in preclinical models and therefore do not directly establish clinical efficacy in patients. Second, many findings are based on transcriptomic inference, including pathway scoring, ligand–receptor analysis, and predicted regulatory networks, and require functional validation. Third, translation to human stroke is complicated by patient heterogeneity, including age, sex, vascular risk factors, comorbidities, infarct size, stroke subtype, treatment timing, and reperfusion status. Future studies incorporating human samples, longitudinal designs, spatial transcriptomics, and experimental validation will be essential to determine whether mechanisms identified by sc/snRNA-seq could inform patient stratification, therapeutic target selection, and treatment optimization in ischemic stroke.

## 5 Conclusion

In conclusion, sc/snRNA-seq has advanced ischemic stroke research by enabling high-resolution dissection of cellular heterogeneity, dynamic state transitions, and molecular networks that shape stroke injury and recovery. These technologies have moved the field beyond bulk tissue-level observations by revealing cell-type-specific responses across immune, vascular, glial, neuronal, and peripheral compartments. They have also provided new insights into key pathological and reparative processes, including neuroinflammation, BBB disruption, cell death, metabolic dysregulation, aging-related vulnerability, neuroregeneration, remyelination, and therapeutic response. As sc/snRNA-seq technologies continue to mature, their integration with spatial transcriptomics, multi-omics profiling, longitudinal sampling, and functional validation will be essential for translating descriptive atlases into mechanistic and therapeutic discoveries. Such approaches may allow more precise mapping of stroke biology across disease stages, stroke subtypes, affected brain regions, systemic organs, and clinical complications. Single-cell technologies may help identify actionable cellular targets, define therapeutic windows, stratify patient subgroups, and guide the development of more precise, mechanism-based interventions. By linking cellular mechanisms to therapeutic opportunities, sc/snRNA-seq is poised to play an increasingly key role in reshaping the future of ischemic stroke research and treatment.
